# Appendix to the Report of the London Committee of Apothecaries and Surgeon-Apothecaries

**Published:** 1813-11

**Authors:** 


*For the Medical and Physical Journal.*

APPENDIX *to the* REPORT *of the* LONDON COMMITTEE *of* APOTHECARIES *and* SURGEON-APOTHECARIES.

(Continued from p. 315.)

No. I.

*Copy of a Letter directed to Sir Francis Milman, Bart. President, &c. and to the Fellows of the Royal College of Physicians.*

GENTLEMEN,

THE Committee appointed by a General Meeting of the Apothecaries of England and Wales, held at the Crown and Anchor Tavern, on Friday, the 20th of November, 1812, to carry into effect certain resolutions then agreed to, beg leave to transmit to you a copy of their report, with the resolutions subjoined, as approved of by that meeting.

The Committee are of opinion that the management of the sick should be as much as possible under the superintendance of the physician; but as in a very great majority of cases the assistance and skill of the apothecary must be relied upon, no one should be allowed to practise as an apothecary without having previously submitted to a suitable examination.

The Committee suggest that there be a distinct privileged body established by the authority of parliament for such examination, and to superintend the general professional interests of apothecaries and surgeon-apothecaries throughout England and Wales, insuring by this means the better preservation of the public health. By the authority to be vested in the proposed superintending body, the apothecary will be required to be universally well qualified, and be thus rendered more worthy of public confidence. The Committee, therefore, submit, that he should possess a legal claim to moderate remuneration for his attendance and professional skill, under such modifications as may hereafter be judged proper.

The Committee are satisfied that any measures which may be considered as calculated to promote their views, must be confirmed by an act of the legislature, which will, unavoidably, be attended with considerable expence. They have, consequently, made it a primary object, and provided such a fund, as will, in their estimation, be adequate to that purpose.

The Committee are desirous to obtain the sanction and concurrence of the legally-constituted bodies of the profession; and they wish it to be distinctly understood, that they are extremely anxious that the regulations to be proposed shall, in no degree, interfere with their established privileges.

They have, therefore, determined to address the Royal College of Surgeons, and the Society of Apothecaries, at the same time with yourselves; and trust that they shall receive your countenance and support in a petition to parliament for the protection and regulation of the practice of the apothecary.

(Signed by desire of the Committee)

*Bloomsbury-square,* G. M. Burrows, Chairman.
*Dec.* 11, 1813.

*Note.*—A similar letter was addressed to Thompson Forster, Esq. Master, &c. and to the Governors and Court of Assistants of the Royal College of Surgeons: and another with the latter paragraph omitted, and the following paragraph graph substituted, to the Master, Wardens, and Court of Assistants, of the Society of Apothecaries.

"They have, therefore, determined to address the Royal College of Physicians and Surgeons at the same time with yourselves.

But as the major part of the members of your society have the same general interests as other apothecaries, and can appreciate more correctly the extent of their grievances, the Committee trust their application to you will be favorably received and countenanced.

With such approbation they will be enabled more confidently to call upon the Royal College of Physicians and Surgeons, to unite in a petition to parliament for the protection and regulation of the practice of the apothecary.

(Signed by the desire of the Committee)

*Bloomsbury-square,* G. M. Burrows, Chairman."
*Dec.* 11, 1812.

---

No. II.

(Copy.) *Bloomsbury-square,*

*Dec.* 25, 1812.

SIR,

As Chairman of the Committee elected by a General Meeting of the Apothecaries of England and Wales, I was instructed to direct the report and resolutions of that meeting, with a letter from the Committee, to the President and Fellows of the Royal College of Physicians.

Conformably with those instructions, I had the honor to send to you, as the president, the printed report of the meeting, and the letter of the Committee, signed by me as chairman, on the 11th instant, for you, officially, to submit to the fellows.

It has been intimated, that my letter, with the report, may be considered by the College a private communication to you; but though this appears to me improbable, yet, to prevent so erroneous an impression, I take the liberty of addressing you again as president, to request that you will lay the said letter, with the report, before the Royal College of Physicians at their next meeting, and that you will condescend to give orders that the Committee be favored with an early answer.

I have the honor to be, Sir,
Your obedient humble Servant,
G. M. Burrows, Chairman.

*To Sir Francis Milman, Bart.*

No.

No. III.

SIR, *Brook-street, Dec. 25, 1812.*

I had the honor of your letter of the 11th, and I have this moment received your favor of this day's date. I understood your first letter to have been only a circular form, of which you had addressed copies to a considerable number of my brethren, as you expressed a determination to address the Royal College of Surgeons and the Society of the Apothecaries at the same time with the College of Physicians. I waited for the execution of this declared purpose, and expected to receive your intended formal application, with your commands to lay it before the College, which I would have obeyed with great pleasure on the 22d of this month, the usual day of our meeting. No such document having reached me, I thought it possible the Committee might have altered the resolution they had formed; and not being particularly desired to do so, I did not deem myself justified in laying your first letter before the gentlemen assembled on the 22d in Warwick-lane. Should it continue to be the wish of your Committee, I will take the first opportunity of a meeting of the College to lay your letters before them.

I have the honor to be, Sir,

Your most obedient humble Servant,

*To G. M. Burrows, Esq.* F. MILMAN.
*Bloomsbury-square.*

---

No. IV. *Bloomsbury-square,*

SIR, *Dec. 26, 1812.*

Accept my acknowledgments for the distinguished attention with which you honored my letter of yesterday, by your answer of the same date.

The letter of the 11th instant, which I subscribed and sent by desire of the Committee of Apothecaries, was directed to the President and Fellows of the Royal College of Physicians. The Committee, therefore, could not conceive it essential to request a letter to be laid before your learned colleagues, which was expressly addressed to them as well as to you, sir, their president; and which they consequently imagined would be presented, as a matter of course, at the meeting of the College on the 22d of December.

The report was sent as a mark of respect to every member of the executives of the medical bodies to whom the Committee were directed to apply, by the fifth resolution of the General Meeting of Apothecaries, held November 20th; but the letter of the Committee was intended as the specific application, and was particular, and not general or circular.

May I take the liberty of soliciting that you will be pleased to inform me when the meeting of the College will take place, to which you propose submitting the letter of the 11th instant, that I may communicate your reply to the Committee?

I have the honor to be, Sir,
Your obedient humble Servant,
G. M. BURROWS, Chairman.

*To Sir Francis Milman, Bart.*

---

No. V. *Bloomsbury-Square,*

*Dec.* 26, 1812.

SIR,

The letter from the Committee of Apothecaries subscribed by me, dated December 11th, and addressed to you as the Master, and to the Governors and Court of the Royal College of Surgeons, I request you will lay before the College, at their first meeting, if that be not already done; and I further take the liberty to express my hope, that you will return an answer as soon as the necessary forms admit.

I have the honor to be, Sir,
Your obedient humble Servant,
G. M. BURROWS, Chairman.

*To Thompson Forster, Esq.*
*Master of the Royal College of Surgeons.*

---

No. VI.
*Southampton Street, Bloomsbury,*
*December* 28, 1812.

Mr. Forster's respects to Mr. Burrows, and begs leave to mention that he laid his letter from the Committee of Apothecaries before the Court of Examiners on the 18th instant; that Court not being competent to the taking it into consideration, it was referred over to the first General Court of Assistants of the College of Surgeons.

*To G. M. Burrows, Esq.*
*Chairman of the Committee of Apothecaries.*

---

No. VII. *Bloomsbury Square,*

*Jan.* 1, 1813.

SIR,

The Committee of the Apothecaries of England and Wales have been made acquainted with the correspondence which has taken place with you, relative to their letter of the 11th of December, directed to the President and Fellows of the Royal College of Physicians.

I have the honor to inform you, that they are extremely concerned to find it was not presented at their meeting on the 22nd, agreeably to their intentions. The Committee therefore make it their particular request, that it may be submitted to the College.

As

As I have not yet been favored with an answer to the question with which I closed my letter of the 26th, the Committee beg leave most respectfully to suggest their wish, that you may think it right to call a meeting of the College at an early period, for the purpose of taking their letter of the 11th into consideration.

The Committee, with great deference, venture this proposal; but further delay will be attended with material inconvenience, as it may prevent the passing of a Bill through Parliament during the present Session.

I have the honor to be, Sir,

Your obedient humble Servant,

*To Sir F. Milman, Bart.* G. M. Burrows, Chairman.

(To be continued.)

